# Nei-like 1 (NEIL1) excises 5-carboxylcytosine directly and stimulates TDG-mediated 5-formyl and 5-carboxylcytosine excision

**DOI:** 10.1038/s41598-017-07458-4

**Published:** 2017-08-21

**Authors:** Anton Slyvka, Karolina Mierzejewska, Matthias Bochtler

**Affiliations:** 1 International Institute of Molecular and Cell Biology, Trojdena 4, 02-109 Warsaw, Poland; 2 Polish Academy of Sciences, Institute of Biochemistry and Biophysics, Pawinskiego 5a, 02-106 Warsaw, Poland

## Abstract

Thymine DNA glycosylase (TDG) and Nei-like 1 (NEIL1) have both been implicated in the base excision repair step of active DNA demethylation. The robust glycosylase activity of TDG on DNA substrates containing 5-formylcytosine (5fC) or 5-carboxylcytosine (5caC) is universally accepted, but the mode of action of NEIL1 is still debated. Based on genetic experiments, it has been suggested that NEIL1 acts redundantly with TDG and excises 5fC and 5caC directly. However, this result has been disputed, and it was suggested instead that NEIL1 is recruited by the monofunctional TDG for the 2′-deoxyribose excision step. Using purified human NEIL1 and its catalytically impaired P2T and E3Q variants as controls, we detect NEIL1 activity on 5caC, but not a 5fC containing dsDNA substrate. We confirm direct NEIL1 TDG binding and NEIL1 mediated 2′-deoxyribose excision downstream of TDG glycosylase activity. NEIL1 acts not only downstream of TDG, but also enhances TDG activity on 5fC or 5caC containing DNA. NEIL1 mediated enhancement of the TDG glycosylase activity is substrate specific and does not occur for dsDNA with a T/G mismatch.

## Introduction

Active DNA demethylation takes place in the absence of DNA replication. In animals, the process involves modification of the methylated base or a nearby base, either by deamination through AID^1, 2^ or by oxidation by the ten eleven translocation (TET) α-ketoglutarate dependent dioxygenases^3, 4^. The modified bases are then replaced by DNA damage repair pathways^5–7^. Despite some evidence for the possible involvement of nucleotide excision repair (NER)^8^ and non-canonical mismatch repair (ncMMR)^9^ in this process, the bulk of repair in active DNA demethylation seems to occur by the base excision repair (BER) pathway^10, 11^. Although active DNA demethylation transiently breaks DNA strand continuity, the process avoids formation of DNA single and double strand breaks with surprising efficiency^7^.

Uracil and thymine, the deamination products of cytosine and 5-methylcytosine (5mC), can be excised by UNG2, SMUG1, TDG, MBD4^12^ and by TDG and MDB4^13^, respectively. The fate of oxidized 5-methylcytosine (5mC) bases in active DNA demethylation is less clear. TETs oxidize 5mC to 5-hydroxymethylcytosine (5hmC)^3^ and then further to 5-formylcytosine (5fC) and 5-carboxylcytosine (5caC)^4^. 5hmC bases are not excised by any of the base excision repair glycosylases, despite an intriguing report that NEIL1 binds better to DNA containing 5hmC containing than unmodified DNA^14^. In contrast to 5hmC, both 5fC and 5caC can be efficiently processed by TDG^15^, and possibly other base excision repair glycosylases^7^.

Thymine DNA glycosylase (TDG) is monofunctional. It catalyzes only the excision of modified or mismatched DNA bases. For the excision of the 2′-deoxyriboside (and the subsequent downstream steps for nucleotide replacement) TDG has to rely on other enzymes of the BER pathway^16, 17^. TDG is essential, and loss of *Tdg* is embryonically lethal, at least in the mouse. Interestingly, abnormalities in *Tdg* null mice appear very abruptly. Until about day 10.5 dpc, embryos appear normal, but then they die within the next two days^18, 19^. TDG is active in the repair of damage from hydrolytic deamination of aminopyrimidines^16, 17^. The enzyme has been named for its activity against DNA containing thymines mispaired with guanines, which arise from spontaneous, methyltransferase-promoted, or AID catalyzed 5mC deamination. TDG activity is cell-cycle dependent. TDG is present in G1. Cells entering S-phase eliminate TDG through the ubiquitin-proteasome system and do not restore TDG levels until G2^20^. The degradation can be triggered at the replication fork. TDG possesses a PIP degron, which directs ubiquitination under the control of a ligase and PCNA^21, 22^. Depletion of TDG in S-phase is thought to prevent excision of T from T/G mismatches during a time when these may be due to polymerase errors and when repair of a T/G mismatch should involve repair of the daughter strand, irrespective of base identity.

Evidence for a role of TDG in oxidation-mediated active DNA demethylation has accumulated in recent years. Biochemical activity of TDG against 5fC and 5caC containing DNA has been demonstrated in several independent studies^10, 15^. Interestingly, TDG excises 5fC and 5caC opposite G at higher rate than T opposite G^15^, supporting the notion that this is a physiologically relevant activity. Rescue of gene expression of a methylated and TET oxidized plasmid was severely impaired in TDG deficient cells, but could be restored by expression of either wild-type TDG, or at least partially by a TDG mutant retaining 5caC excision activity^23^. Comparisons of gene expression patterns in *TDG* null and control cells suggests TDG contributes to the maintenance of active and bivalent chromatin throughout differentiation, by countering aberrant *de novo* methylation^18^. Moreover, Tdg is necessary for demethylation during the mesenchymal to epithelial transition (MET)^24^, which in turn is required for organogenesis and somitogenesis. However, TDG is not universally required for active DNA demethylation. Surprisingly, *Tdg* is barely expressed in the oocyte and zygote and is not required for demethylation of the paternal pronucleus^25^. The unexpected dispensability of TDG in some active demethylation events has triggered the search for other DNA repair enzymes, particularly from the BER pathway, that may take part in active DNA demethylation. Screens in cell culture indicate possible roles of UNG2^26^, and of the Nei-like enzymes^23^. However, TDG remains the only glycosylase for which activity against 5fC and 5caC in DNA has been convincingly demonstrated *in vitro*.

Nei-like 1 (NEIL1) is a bifunctional DNA glycosylase that catalyzes both excision of the modified base (by a hydrolysis reaction) and the subsequent excision of the 2′-deoxyribose, by a β-,δ-lyase reaction that ultimately that breaks the DNA strand and leaves behind phosphorylated DNA ends. The δ-elimination step requires prior β-elimination^27^. Therefore an abasic site can appear as an intermediate before formation of the phosphorylated 3′-end of the DNA. The active site of NEIL1 is known from biochemical and crystallographic studies^28, 29^. Mutations P2T and E3Q in the active site drastically reduce or abolish NEIL1 activity in a wide range of species^30, 31^. NEIL1 is a typical S-phase protein involved in pre-replicative repair of oxidized bases in the human genome^32^ and is found in direct association with the replisome^33, 34^. *In vivo*, loss of *NEIL1* is better tolerated than loss of *TDG*. *NEIL1* null mice are viable, but develop metabolic syndrome, a combination of severe obesity, dyslipidemia, fatty liver disease, and a tendency for hyperinsulinemia^35^. NEIL1 excises oxidized pyrimidines in DNA. Substrates include DNA containing oxidized hydantoin lesions, oxidized pyrimidines, formamidopyrimidines, thymine residues oxidized at the methyl group, and stereoisomers of thymine glycol^36^. NEIL1 substrate specificity can be altered by ADAR1 catalyzed RNA editing. The unedited and edited mRNAs are translated to enzymes with lysine and arginine (K242 and R242, numbering for the human enzyme) forming a side wall of the active site pocket for the substrate base^37^. NEIL1 has been described as active on single-stranded DNA and on DNA of bubble or forked structure, but other work suggests that it excises lesions more efficiently in single- than double-stranded DNA. NEIL1 can bind directly to proliferating cell nuclear antigen (PCNA) and travels with the replication fork (24). Apart from its role in the repair of oxidative DNA damage, the enzyme has been linked to DNA demethylation by at least two independent studies that suggest different models for its mode of action^23, 38^.

Leonhardt and coworkers have provided evidence for a possible role of NEIL1 (and the paralogues NEIL2 and NEIL3) in active DNA demethylation^23^. They have shown that NEIL1 co-localizes with TET proteins in a three hybrid assay. In the same work, they have also shown that wild-type cells can rescue expression of a reporter protein from a plasmid that has been subjected to *in vitro* CpG methylation and TET mediated oxidation, but not a plasmid subjected only to *in vitro* methylation. The rescue of gene expression from the methylated and oxidized plasmid was severely impaired in TDG deficient cells, but could be restored by expression of either wild-type TDG, or at least partially by NEIL1 or a TDG mutant retaining 5caC excision activity (TDG N168D). As NEIL1 reduced the levels of 5fC and 5caC, but not 5hmC, in a TDG null background, they concluded that NEIL1 can act redundantly with TDG and excise 5fC or 5caC from DNA^23^.

Niehrs and coworkers found NEIL1 and NEIL2 in a knockdown screen of candidate glycosylases looking for impaired replacement of 5fC or 5caC in test DNA in HeLa cell extracts^38^. In their hands, biochemical tests indicated that NEIL1 (and NEIL2) did not preferentially bind to 5hmC, 5fC or 5caC, and that neither enzyme could excise 5hmC, 5fC or 5caC in DNA, even though the enzymes were active on a control substrate DNA with 5-hydoxyuracil lesion. Based on their finding of a physical interaction between NEIL1 and TDG, Niehrs and coworkers have proposed that NEIL1 (and NEIL2) do not process oxidized 5-methylcytosines directly, but cooperate with TDG in the processing of abasic sites generated by 5caC excision. According to this model, the monofunctional glycosylase TDG excises the 5caC, recruits NEIL1 (or NEIL2) by direct physical interaction, and is then displaced by NEIL1 or NEIL2, which then catalyze the excision of the deoxyribose^39^.

Using recombinant human TDG and NEIL1 (in the edited form with R242 instead of K242), or variants of these enzymes (Suppl. Fig. 1), we have carried out biochemical experiments to characterize the direct and indirect roles of NEIL1 in the excision of 5fC and 5caC. In agreement with data by Leonhardt and co-workers^23^, we find that NEIL1 alone is active on 5caC containing DNA, although at a lower level than TDG. We confirm cooperation between NEIL1 and TDG^39^, and also stimulation of the glycosylase activity of TDG by NEIL1^39^. We show for the first time that stimulation of TDG by NEIL1 does not require the glycosylase or lyase activites of NEIL1, and occurs specifically for demethylation intermediates 5fC and 5caC, whereas the enzyme, at least in high concentration, inhibits TDG activity against substrates containing a T/G mismatch. We also demonstrate that stimulation between NEIL1 and TDG is reciprocal, because TDG promotes the lyase activity of NEIL1 on DNA containing an abasic site.

## Results

### Activity of TDG against 5caC/G, 5fC/G and T/G containing substrates

TDG is a monofunctional glycosylase. We tested the activity of full length TDG against DNA substrates containing either 5fC or 5caC or T opposite a G base. The strand containing 5fC, 5caC or T was radiolabeled using T4 polynucleotide kinase (PNK), and then annealed to a non-radiolabeled complementary strand with a G opposite the 5fC, 5caC or T. The reaction was carried out with an excess of enzyme over DNA. Abasic (apurinic, apyrimidinic, AP) sites generated by the enzymatic reaction were then converted to single strand breaks by alkaline treatment, and reaction products were analysed by gel electrophoresis under denaturing conditions, followed by autoradiography. At pH 7.5, we observed robust glycosylase activity against DNA substrates containing 5fC and 5caC that exceed the activity against a substrate with T/G mismatch (Suppl. Fig. 2A). Due to the instability of full-length TDG protein against degradation, which leads to inhomogeneity in the sample, we separately expressed the much more stable catalytic core region of TDG^40^, which we termed TDGcd and tested it on the same substrates. We observed slightly weaker, but still robust activity (Suppl. Fig. 2B). The activity was completely lost when the experiment was repeated using the N140A variant, excluding a contaminant as the source of the activity. The activity was highest for the 5caC 2′-deoxynucleotide in the context of double stranded DNA, slightly weaker in the context of single stranded DNA, and much weaker when 5caC was present in a bubble (Suppl. Fig. 3).

### Activity of NEIL1 against 5OHC/G

NEIL1 is a bifunctional glycosylase that among other substrates acts on dsDNA containing 5-hydroxycytosine (5OHC) sites. For the human enzyme, the NEIL1 P2T and E3Q mutations are known to suppress mostly or completely the generation of DNA strand breaks at a thymine glycol site^31^. However, the published data do not indicate whether only one or both of the activities were affected by the mutations. In order to measure only the glycosylase of NEIL1 activity, independently of the downstream β- and δ-lyase reactions, we carried out the NEIL1 assay as for TDG with work-up in alkaline conditions to convert AP-sites to single strand breaks. With alkaline workup, we observed robust cleavage of the 5OHC containing DNA strand by the wild-type enzyme. The P2T mutant was impaired for the glycosylase reaction, but nevertheless retained some weak glycosylase activity on the 5OHC substrate. In contrast, the E3Q variant of the enzyme almost completely abolished this activity (Suppl. Fig. 4A). In order to measure only the lyase activity of NEIL1 and variants, we used a dsDNA substrate with an abasic site. As abasic sites are prone to spontaneous strand breakage (especially in alkaline conditions), we did not use 2′-deoxyoligonucleotides containing a chemically incorporated abasic site, but rather generated the abasic site using TDG, which was subsequently removed by phenol-chloroform extraction. The AP-site containing DNA was incubated with NEIL1 or its variants and then analysed without alkaline workup. We observed a robust strand cleavage. With the wild-type enzyme, the reaction proceeded essentially to completion by δ-elimination. The P2T mutant had drastically impaired lyase activity, as only small amounts of β-elimination product were observed. In contrast, the E3Q variant of NEIL1 retained some residual lyase activity, judging from the observation of both β- and δ-elimination products (Suppl. Fig. 4B). We conclude that the P2T and E3Q amino acid exchanges affect human NEIL1 in a very similar way as *E. coli* Nei^30^.

### Activity of NEIL1 against 5caC/G, but not 5fC/G

**Figure 1:**
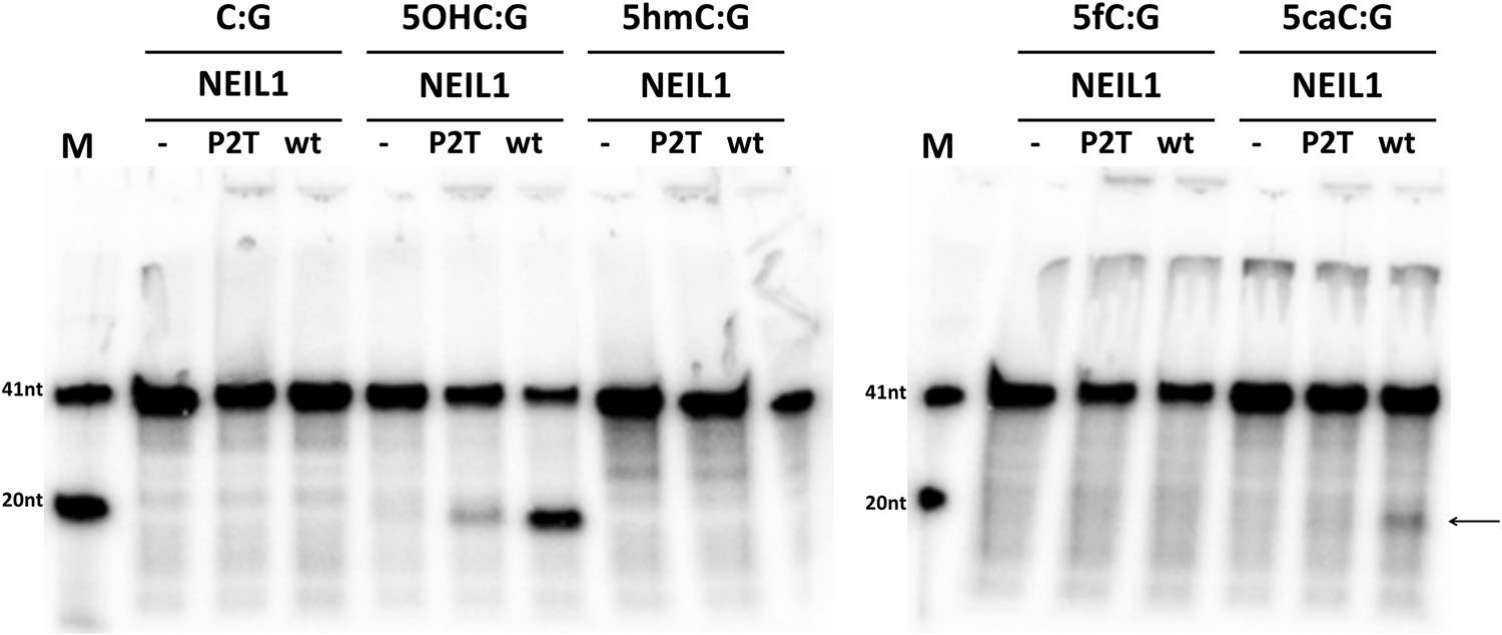
NEIL1 can excise 5caC from DNA: The glycosylase activities of NEIL1 or the control P2T variant were tested using dsDNA substrates (200 nM enzyme, 20 nM dsDNA). The ^32^P radiolabelled strand contained the indicated base in position 20, placed opposite a G base in the complementary strand. After the enzymatic reaction (37 °C, 60 min), samples were subjected to alkaline conditions to break DNA strands at AP-sites. DNA was analysed by denaturing gel electrophoresis and visualized by autoradiography. The 5′ cleavage product of NEIL1 glycosylase activity on the 5caC containing DNA is marked by an arrow. In this and the following figures, “-” designates the absence of enzyme.

**Figure 2:**
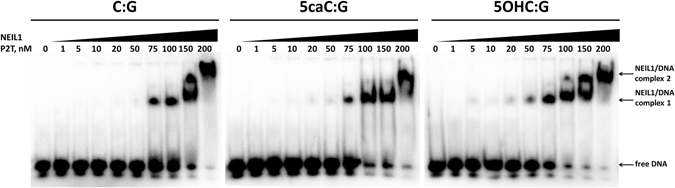
NEIL1 binds better to 5caC or 5OHC containing DNA than unmodified DNA: The electrophoretic mobility of 10 nM radiolabeled dsDNA containing either only unmodified bases, a 5caC base, or a 5OHC base was monitored in native conditions in the presence of increasing amounts of the inactive NEIL1 P2T variant (to prevent DNA cleavage during the experiment) by autoradiography. We have observed the formation of NEIL1/DNA complex (1) and supercomplex (2) at lower protein concentration with an oligo containing 5OHC:G (positive control) or 5caC:G (experiment) than in case of the oligo containing C:G. Moreover we can also observe the disappearance of free DNA that contained 5OHC:G and 5caC:G at lower NEIL1 P2T concentration than in case of DNA containing C:G.

Next, we tested potential glycosylase activity of NEIL1 on intermediates of oxidation based DNA demethylation, and on DNA containing a 5OHC site as a control (Fig. 1). All substrates were tested in the context of double stranded DNA, again with excess of enzyme over DNA to measure catalytic rates. NEIL1 showed the expected robust activity against the 5OHC containing substrate. However, NEIL1 did not cleave 5hmC, despite the identification of NEIL1 as a 5hmC binding protein in a large scale screen^14^. We detected no or at most an extremely faint activity of NEIL1 against DNA containing 5fC. In contrast, DNA that contained 5caC opposite G in double stranded DNA was cleaved by NEIL1, but not the glycosylase defective NEIL1 P2T (Fig. 1) or E3Q variants (Suppl. Fig. 5). The P2T variant (the E3Q variant was not tested) shifted double stranded (ds) DNA containing 5OHC or 5caC slightly better than unmodified control dsDNA of the same sequence in an electrophoretic mobility shift (EMSA) experiment (Fig. 2), as predicted based on the observed activity. Taken together, our data support the conclusion of the Leonhardt group that NEIL1 can act directly and alone on DNA containing 5caC. However, the glycosylase activity of NEIL1 against DNA containing 5caC is weak compared to the activity of TDG, most likely explaining why it was not detected in a previous biochemical assay.

### Structural plausibility of a direct activity of NEIL1 against 5caC containing DNA

**Figure 3:**
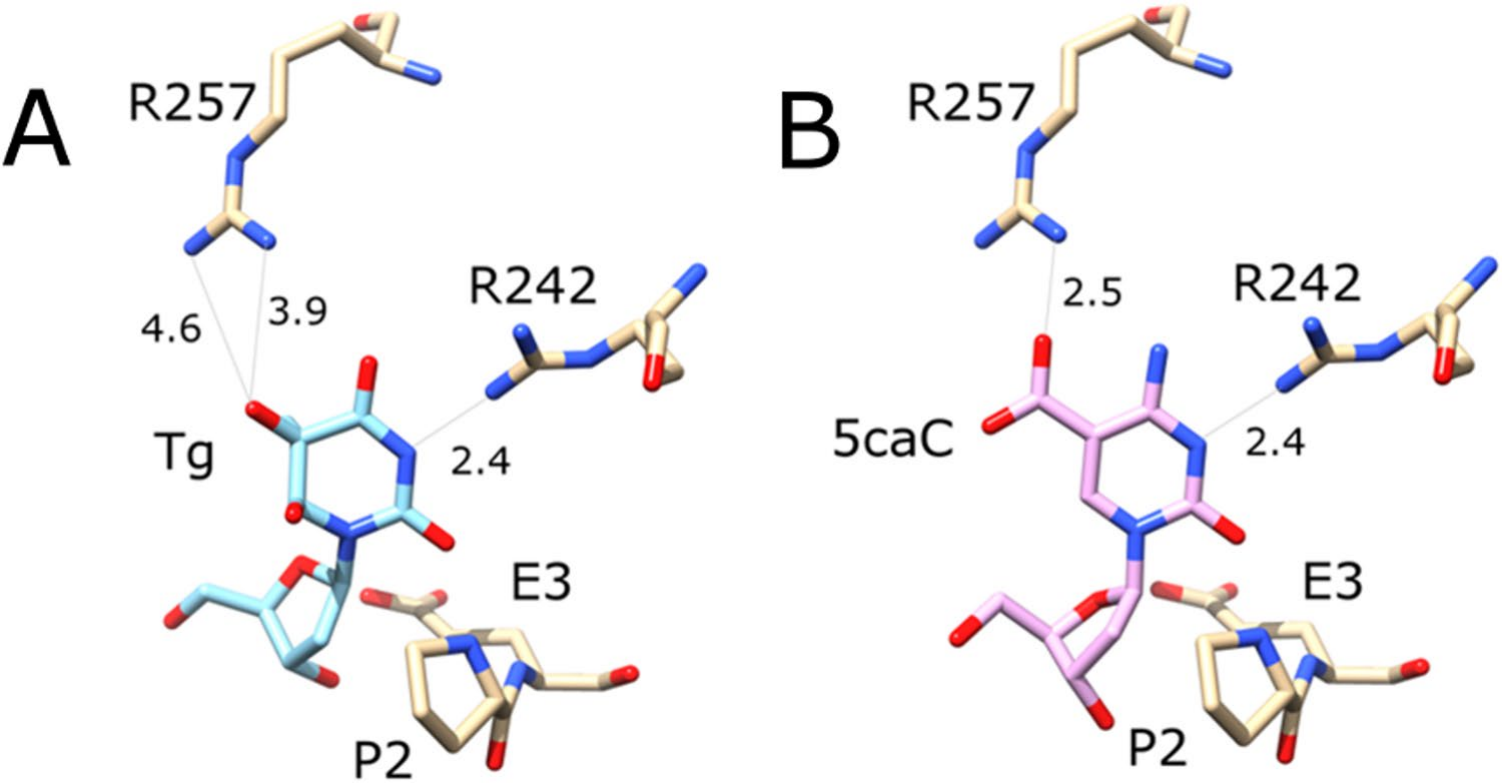
Structural plausibility of the NEIL1 glycosylase activity against DNA containing 5caC: (**A**) Composite model of the active site with flipped 2′-deoxythymidine glycol. The figure was drawn based on the coordinates determined by Zhu and coworkers for the P2G variant (PDB accession 5ITY)^29^. The coordinates for the P2 residue in the wild-type were taken from the structure of NEIL1 with a THF abasic site analogue (PDB accession 5ITT)^29^. Distances in panel A are experimental distances for the molecule A in the structure 5ITY. Both arginine residues adopt different conformations in the other molecules of the asymmetric unit. Note that the pocket for the base is completely obliterated in the 5ITT structure with abasic site analogue. (**B**) Composite model of the active site with flipped 5caC 2′-deoxynucleotide. The 5caC 2′-deoxynucleotide was taken from the structure of TDG with DNA containing 5caC (PDB accession 3UO7)^53^, and placed based on least squares superposition of the (planar) 5caC base on the (non-planar) thymidine glycol base. Note that Tg has been reported to adopt a lactim tautomeric structure with hydrogen bond acceptor in the N3 position^29^. 5caC also has a hydrogen bond acceptor in this position, and is likely protonated in this position to support departure of the base, as suggested for TDG before^45^. The catalytic residues P2 and E3 are strictly conserved. The position of R242 is subject to RNA editing, making this residue either R or K^37^. R257 is conserved in many, but not all vertebrates. Numbers indicate distances in Å.

Several crystal structures of human NEIL1 have been reported, including one of the edited from of NEIL1 (containing R242 instead of K242) in complex with DNA containing thymine glycol^29^. The crystal structure confirms that NEIL1, like other DNA glycosylases, flips the substrate base from the DNA stack and accommodates it in a pocket of the enzyme^29^. The thymine glycol has been reported to adopt a lactim form with N3 accepting a hydrogen bond from arginine R242 (Fig. 3A). Based on least squares superposition of the ring atoms of thymine glycol and 5caC, we have constructed a model of the Michalis complex of NEIL1 and a DNA substrate containing 5caC DNA (Fig. 3B). The model shows that the NEIL1 base is sufficiently spacious to accommodate the 5caC base. Cytosine has a hydrogen bond acceptor at the N3 position, and can therefore also form the hydrogen bond to R242 (and perhaps also K242 in case of the non-edited form of NEIL1). Moreover, the model predicts that R257 may form a salt bridge with the carboxylate group of 5caC. In case of 5fC, a hydrogen bond may form instead of the salt bridge.

### Mutations to the NEIL1 base binding pocket do not affect activity against 5caC

**Figure 4:**
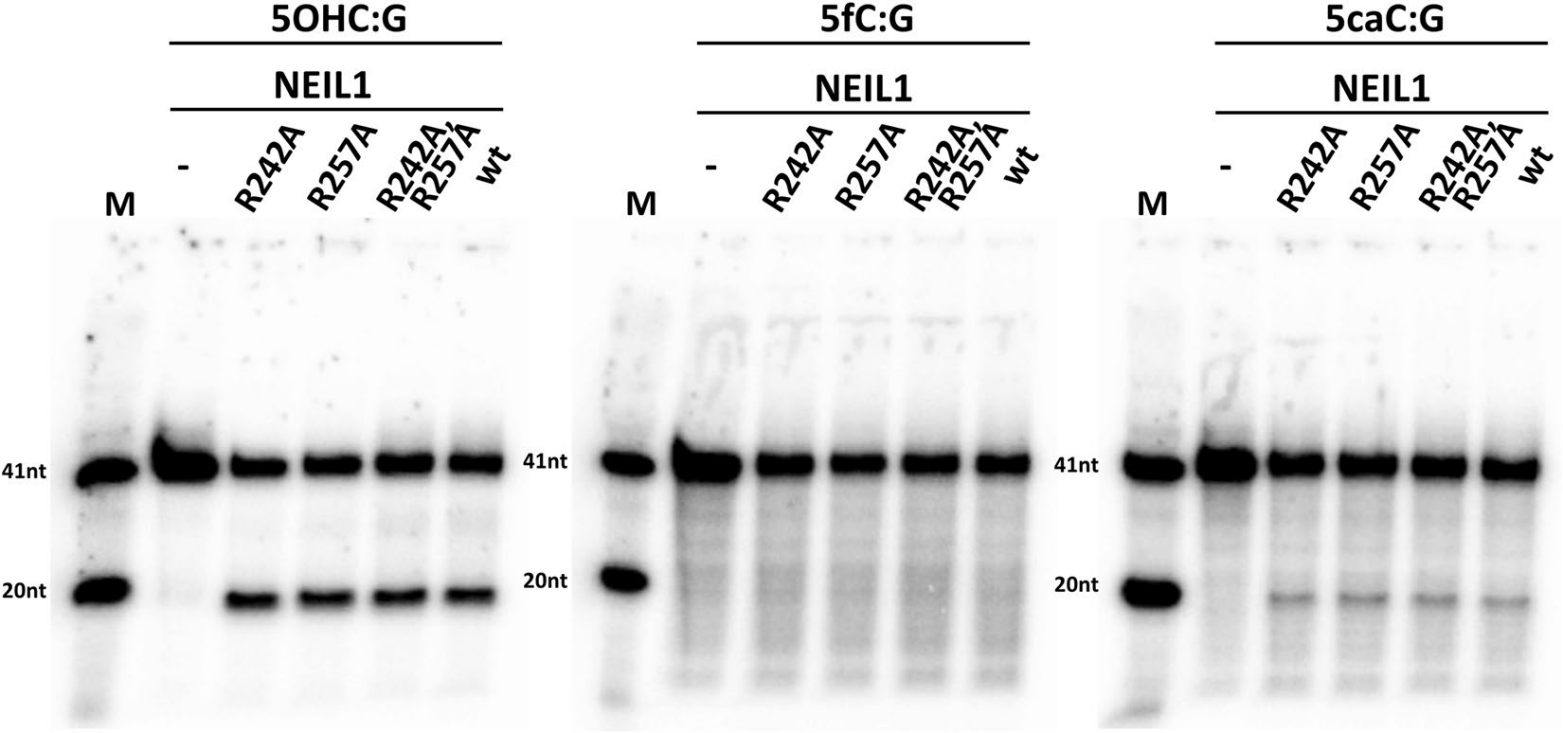
Pocket wall interactions are not required for NEIL1 activity against 5caC: The glycosylase activities of NEIL1 and of the indicated variants were tested by incubation with dsDNA substrates containing a 5OHC, 5fC or 5caC base (^32^P radiolabelled in the modified strand). After 1 hour incubation of 20 nM dsDNA with 200 nM enzyme at 37 °C, the samples were treated with NaOH to induce single strand breaks at abasic sites. Cleavage products were analysed by denaturing gel electrophoresis and visualized by autoradiography.

RNA editing is responsible for the identity of the R242 residue. We checked whether the interaction between this residue and the base was required for 5caC excision. Although protonation of the N3 of the base is thought to weaken the glycosidic bond, the R242A variant of NEIL1 had similar activity against DNA containing 5OHC or 5caC as the wild-type enzyme, and remained inactive against 5fC containing DNA. Next, we tested whether the difference between a possible salt bridge in the case of 5caC and a possible hydrogen bond in the case of 5fC may explain the difference in activity of NEIL1 towards the 5fC and 5caC containing DNA. Surprisingly, this was not the case. The R257A variant of NEIL1, like the R242A variant, exhibited similar activity as the wild-type NEIL1 against both 5OHC and 5caC containing DNA. Even the R242A R257A double mutant was still found to retain the activity. Therefore, it appears that specific interactions between NEIL1 and 5caC play little role in the excision of 5caC from DNA, at least when NEIL1 is present in excess (Fig. 4).

### NEIL1 and TDG bind directly to each other

**Figure 5:**
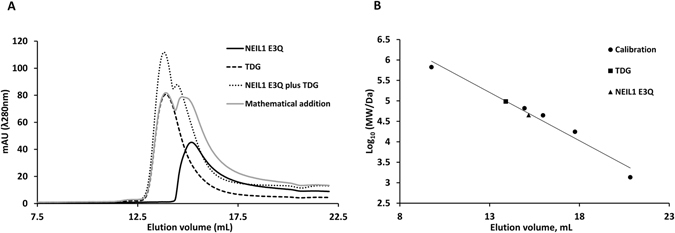
NEIL1 and TDG bind to each other directly: Physical interactions between NEIL1 E3Q and TDG were tested by size exclusion chromatography in SEC buffer (70 mM MOPS, pH7.5, 5% glycerol, 1 mM EDTA, 1 mM DTT and 100 mM KCl). Panel (**A**) shows the chromatograms, panel (**B**) illustrates the calibration of the column. The migration profiles of NEIL1 E3Q (black continuous line) and TDG (black dashed line) in isolation were consistent with monomeric and dimeric states, respectively (the mass of the dimer is used for the TDG data point in the calibration panel). Co-injection of the two proteins (one TDG dimer per NEIL1 monomer) led to an elution profile (grey dotted line) that was significantly different from the sum of profiles for separate injections (grey continuous line).

In thermophoresis experiments, Niehrs and co-workers detected direct physical interaction between NEIL1 and TDG^39^. We tested the interaction using sizing chromatography. Although the molecular masses of TDG (46.1 kDa without tag, 48.4 kDa with tag) and NEIL1 (43.7 kDa without tag, 44.8 kDa with tag) are very similar, the two proteins migrated very differently, because TDG forms dimers, whereas NEIL1 is monomeric. Interaction of TDG and NEIL1 was detectable as a significant deviation of the elution profile for co-injection from the sum of elution profiles for separate injections of the components (Fig. 5).

### NEIL1 and TDG cooperate for the excision of 5caC 2′-deoxynucleoside

**Figure 6:**
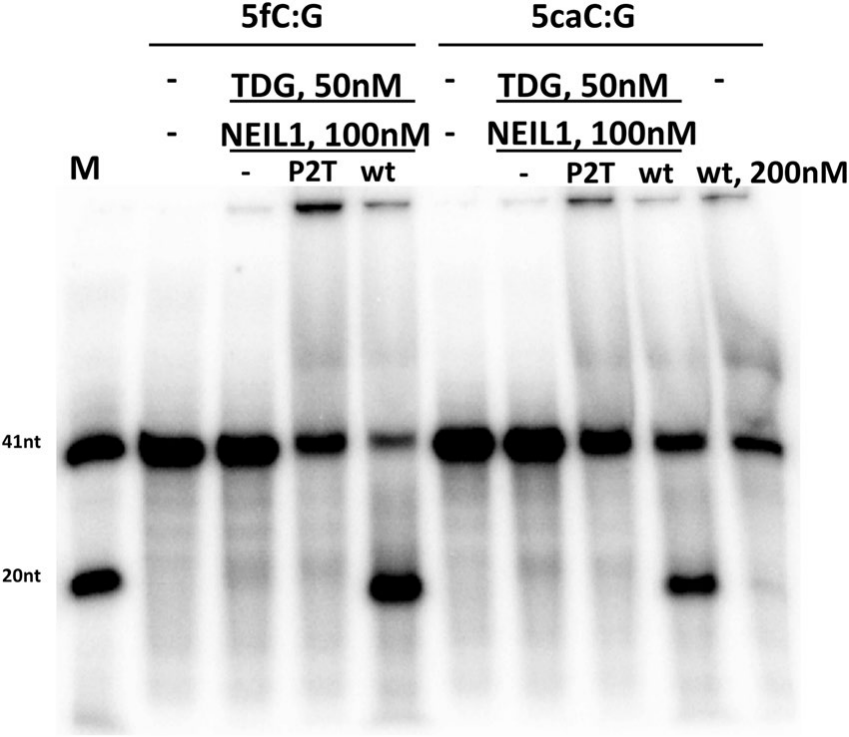
NEIL1 can act downstream of TDG: 50 nM TDG and 100 nM of active NEIL1 or the catalytically impaired NEIL1 P2T variant were incubated (37 °C, 60 min) with 20 nM dsDNA. The ^32^P-radiolabelled strand of the DNA contained at position 20 a 5fC (left) or 5caC (right). Reaction products were not subjected to alkaline conditions and were analysed by denaturing gel electrophoresis and autoradiography. NEIL1 and TDG together catalysed a much greater number of strand breaks than NEIL1 alone. The data show that NEIL1 can efficiently act downstream of TDG to process abasic sites, despite the tendency of TDG to stick to AP-sites^41^.

We first set out to test the model of cooperation TDG and NEIL1 proposed by Niehrs and colleagues. According to their model, the monofunctional glycosylase TDG recruits NEIL1 for excision of the 2′-deoxyribose. We carried out strand break assays with fixed amount of TDG (50 nM) and increasing amounts of NEIL. As substrates, we used duplex DNA containing either 5fC or 5caC in the ^32^P-labelled strand and monitored cleavage of this strand by denaturing gel electrophoresis and autoradiography (Fig. 6). TDG alone or TDG in combination with the catalytically inactive NEIL1 P2T variant did not cause DNA cleavage. However, the wild-type version of TDG in combination with wild-type NEIL1 generated a robust cleavage product. As NEIL1 is not active on 5fC containing DNA, this finding can only be interpreted as NEIL1 processing abasic sites generated by TDG. In the case of 5caC containing DNA, there is a formal possibility that strand breaks could result from the action of NEIL1 alone. However, a control confirmed NEIL1 activity alone could not explain the amount of cleavage product. Thus, our data support the model of TDG-NEIL1 cooperation of Niehrs and coworkers^39^. The data imply that NEIL1 can displace TDG, which otherwise tends to stick to the AP-sites that it generates^41^ (Fig. 6).

### NEIL1 stimulates 5caC and 5fC excision by TDG

Niehrs and co-workers had previously reported that addition of NEIL1 to TDG enhanced the rate of 5fC and 5caC excision over the rate obtained with TDG alone. As they could not detect biochemical activity of NEIL1 alone on DNA containing 5caC, they interpreted this finding as stimulation of TDG 5caC excision activity^39^. In the light of our data on the excision of 5caC by NEIL1 alone, this conclusion required reexamination. We first established that indeed addition of TDGcd to NEIL1 or vice versa increased overall glycosylase activity on a 5caC containing dsDNA substrate (Suppl. Fig. 6).

**Figure 7:**
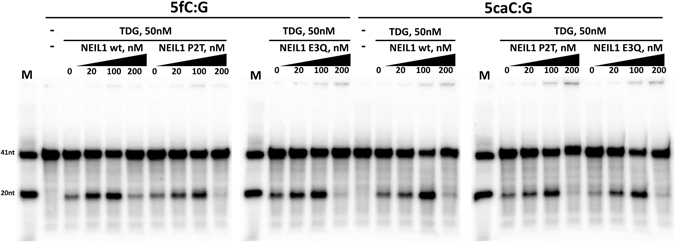
NEIL1 stimulates 5fC and 5caC excision by TDG: 50 nM TDG and varying amounts of NEIL1 or the control P2T variant were incubated (37 °C, 60 min) with 20 nM dsDNA containing a radiolabelled strand with a T base in a T/G mismatch. DNA was then broken at AP-sites in alkaline conditions and analysed by denaturing gel electrophoresis and autoradiography. NEIL1 mediated stimulation of the TDG 5fC and 5caC excision activities does not depend on the NEIL1 catalytic activities.

In order to rigorously check stimulation of TDG glycosylase activity by NEIL1, we incubated a fixed amount of TDG and increasing amounts of not only wt NEIL1, but also of its inactive P2T and E3Q variants, using DNA substrates containing either 5fC or 5caC sites. Reaction products were analysed after workup in alkaline conditions, so that only the glycosylase reaction was measured (Fig. 7). As expected, increasing amounts of NEIL1 led to increasing amounts of strand break product, for both the 5fC and 5caC DNA substrates. At the highest concentration of NEIL1, however, inhibition of glycosylase activity was observed, presumably due to competition between NEIL1 and TDG. Essentially the same increase of glycosylase activity was seen when increasing amounts of the NEIL1 P2T or E3Q were added. The apparently small contribution of NEIL1 glycosylase activity to the overall rate of base excision is consistent with the much lower glycosylase activity of NEIL1 compared to TDG for 5fC or 5caC DNA substrates. At high NEIL1 concentration, inhibition of TDG glycosylase activity was more pronounced for the mutants than for wild-type NEIL1, as would be expected if the inhibition was due to competition in (non-physiological) conditions of limiting DNA. Taken together, our data support the proposed enhancement of TDG glycosylase activity by NEIL1.

### NEIL1 does not stimulate, but rather inhibits TDG activity on a T/G mispair

**Figure 8:**
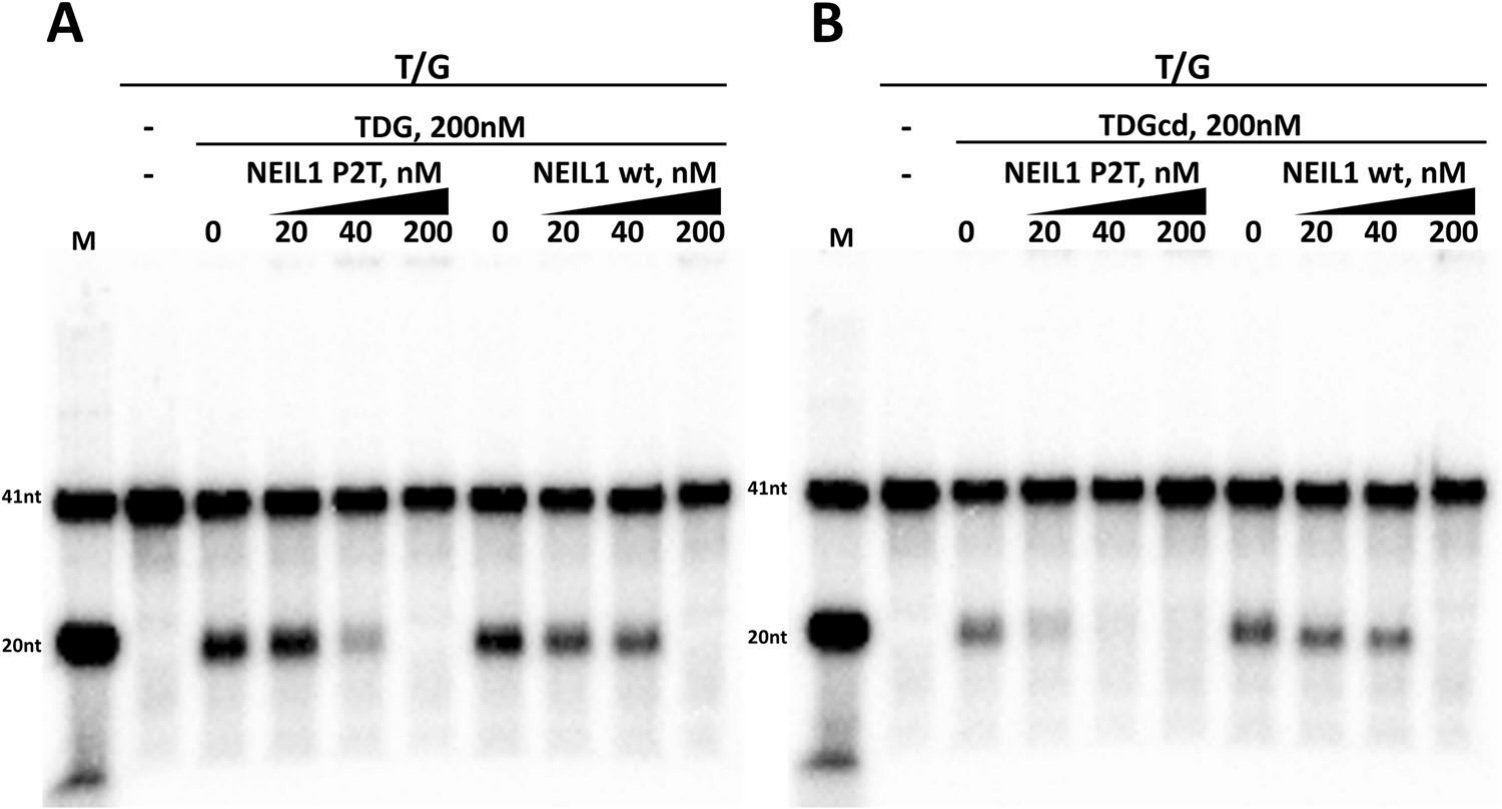
NEIL1 interferes with TDG activity on a T/G mismatch: 50 nM TDG and varying amounts of inactive NEIL1 P2T or NEIL1 wt were incubated (37 °C, 60 min) with 20 nM dsDNA containing a radiolabelled strand with T/G mismatch. DNA was then broken at AP-sites by in alkaline conditions and analysed by denaturing gel electrophoresis and autoradiography. Interference of NEIL1 with TDG glycosylase activity towards the T/G mismatch substrate was independent of the NEIL1 catalytic activities. The NEIL1 inhibits both full-length TDG (**A**) and TDGcd (**B**).

Interestingly stimulation of TDG activity by NEIL1 did not occur for a T/G mismatch substrate. Instead, the observed amount of cleavage product decreased with increasing NEIL1 concentration (Fig. 8A). The same effect was seen when TDG was replaced by TDGcd (Fig. 8B). In both cases, the amount of product decreased already for substoichiometric amounts of NEIL1 compared to TDG or TDGcd (but not DNA). Therefore it is possible that apparent inhibition might be due to competition in conditions of limiting DNA. Hence, we only conclude that the NEIL1 mediated enhancement of TDG glycosylase activity (as seen with 5fC and 5caC DNA substrates) does not occur for a T/G mismatch DNA substrate. At high (local) enzyme concentrations, profound inhibition occurs.

### TDGcd does not stimulate NEIL1 glycosylase activity on 5caC and 5OHC substrates

**Figure 9:**
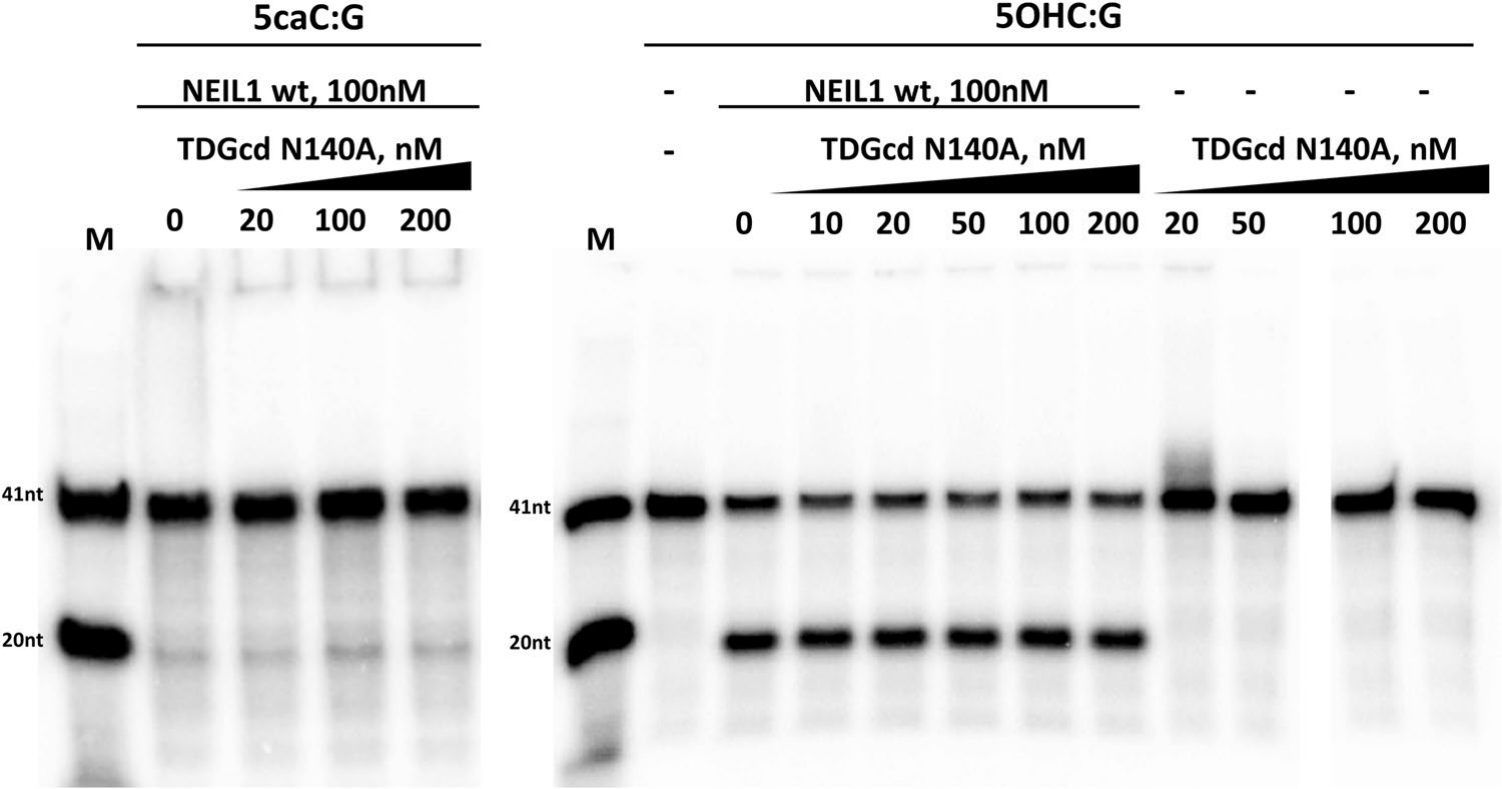
NEIL1 glycosylase activity is not affected by TDGcd: 100 nM NEIL1 and increasing amounts of the catalytically impaired TDGcd N140A were incubated (37 °C, 60 min) with 20 nM dsDNA containing a radiolabelled strand containing either a 5caC or 5OHC base opposite G. After the enzymatic reaction, reaction products were subjected to alkaline conditions to break DNA strands at AP-sites.

Stimulation of the glycosylase activity of TDG and TDGcd by NEIL prompted the question whether stimulation may be reciprocal. If so, then TDGcd should stimulate the glycosylase activity of NEIL1, either on substrates containing DNA demethylation intermediates, or on substrates containing DNA bases damaged by non-enzymatic oxidation. Due to the robust activity of TDG and TDGcd against 5caC substrates, experiments had to be carried out with the catalytically defective N140A variant of TDGcd. NEIL1 glycosylase turned out to be independent of TDGcd, for both the 5caC and the 5OHC substrates (Fig. 9).

### TDGcd stimulates NEIL1 β,δ-lyase activity on an AP-site

**Figure 10:**
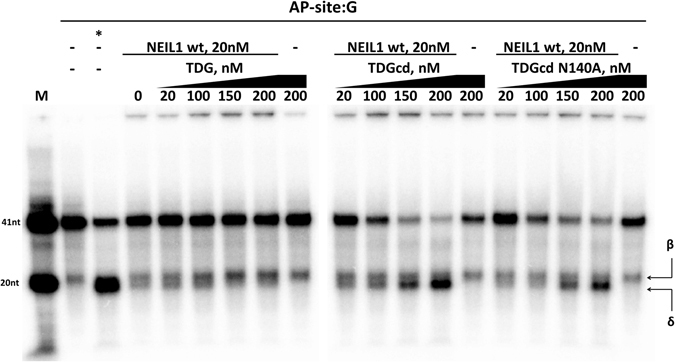
TDGcd or TDGcd N140A stimulate NEIL1 lyase activity: dsDNA containing a ^32^P radiolabelled strand with AP-site in position 20 was freshly prepared by excision of a 5caC base in this position using TDG and by subsequent TDG removal using phenol chloroform extraction. NEIL1 (20 nM) was then incubated with the substrate (10 nM) in the presence of different amounts of TDG, TDGcd or TDGcd N140A (37 °C, 30 min). ‘*’ represents the AP-site containing dsDNA treated with alkaline conditions (positive control). ‘β’ and ‘δ’ mark the products of NEIL1 β- and δ-lyase activities, respectively.

TDGcd (and TDG) excise 5caC from DNA faster than NEIL1. Therefore, overall glycosylase rates would not be significantly increased by (moderate) TDGcd mediated activation of the glycosylase activity of NEIL1. In contrast, enhancement of the NEIL1 lyase activity by TDG would speed up processing of intermediates of oxidative demethylation. In order to test the stimulation of the NEIL1 lyase activity by TDG, we freshly prepared an AP-site substrate TDG excision of 5caC, followed by phenol-chloroform extraction to remove TDG. We then incubated a fixed amount of NEIL1 and DNA (20 nM NEIL1, 10 nM dsDNA) with increasing amounts of either full length TDG, TDGcd, or the inactive N140A variant of TDGcd (Fig. 10). As TDG binds tightly to and dissociates slowly from AP-sites^41^, one may expect suppression of NEIL1 lyase activity by TDG^41^. This is not observed. Full-length TDG affected the NEIL1 catalyzed lyase reaction only weakly, and –if it had any effect– rather increased the amount of the β-elimination product. TDGcd and its inactive N140A variant promoted NEIL1 lyase activity. The significance of the stimulation of NEIL1 lyase activity by TDGcd, but not full-length TDG, is unclear to us. Nevertheless, we can conclude that NEIL1 must be effective at displacing TDG from AP sites (Fig. 10).

## Discussion

### Direct activity of NEIL1 against DNA containing 5caC

The direct activity of NEIL1 against DNA containing 5caC contradicts the report by Niehrs and colleagues^39^, but is consistent with the findings of Leonhardt and coworkers^23^. We considered whether the different conclusions could have to do with RNA editing. Niehrs and coworkers report a lack of activity for the enzyme generated in *Escherichia coli*, which would not be affected by editing^39^, whereas Leonhardt and coworkers report experiments in cell culture^23^, which may be affected by editing. Unfortunately, this explanation for the discrepancy does not seem to hold. Niehrs and coworkers do not comment on the identity of residue 242 in their NEIL1 protein. However, their reference to the ORFeome collection suggests that they have worked with the edited form of NEIL1 with R242 and not K242. Moreover, our own finding that a variant of NEIL1 with A242 retains activity also speaks against editing as a cause of reported discrepancies in NEIL1 activity. Instead, we rather suspect that the NEIL1 activity against 5caC containing DNA is simply too weak to be detected in their assay.

### Size and specificity of the NEIL1 pocket for the excised base

NEIL1 has a spacious pocket for the excised base, as strikingly illustrated by its activity on very bulky ring-opened aflatoxin-deoxyguanosine adducts^42^. An argument for a sufficiently large pocket of NEIL1, which has room for all three atoms of the carboxylate group of 5caC, can be made based on a comparison of known NEIL1 substrates^36^. NEIL1 excises oxidized pyrimidines, thymine glycol, formamidopyridimines, and also 5-formyluracil (5fU)^43^. When glycosidic bonds and bases are overlaid, together the substrates occupy the full space taken by the carboxylate group of 5caC. The conclusion that NEIL1 has space in its base binding pocket for 5caC is also supported by the crystal structures of NEIL1 and homologues^28, 29, 44^. Together, the structures suggest that the pocket for the excised base has substantial plasticity, which may also lead to its occlusion in the absence of substrate.

Our mutagenesis data on the role of R242 and R257 in the excision of the 5-carboxycytosine base suggests that direct interactions between NEIL1 and the excised base have surprisingly mild effects. In particular, the intact activity of NEIL1 R257A against DNA containing 5caC, but not 5fC, rule out the possibility the preference for a salt bridge over a hydrogen bond may explain the ability of NEIL1 to discriminate between 5caC and 5fC. As direct interactions seem to play little role in substrate selectivity, we suspect that glycosidic bond stabilities or spontaneous flipping rates may explain why NEIL1 can excise 5caC, but not 5fC.

### Vulnerability of 5fC and 5caC to glycosylases and NEIL1 activity on 5caC, but not 5fC

The electron-withdrawing properties of formyl- and carboxyl (neutral form) groups have been suggested to promote excision of 5caC and 5fC from DNA by destabilizing the glycosidic bond^45^ and by weakening base pairing between the modified cytosine and guanine bases^46^. It is therefore not surprising that a glycosylase with spacious pocket for the excised base has some activity against DNA containing a 5fC or 5caC base. However, the better activity against 5caC compared to 5fC requires explanation. The 5-carboxyl group (in anionic form, predominating at neutral pH) facilitates N3 protonation, whereas the 5-formyl group hinders it. As N3 protonation strongly destabilizes the glycosidic bond, the effect may account for easier excision of 5caC compared to 5fC. Alternatively, hydration of the formyl group may attenuate the electron-withdrawing properties of the 5-formyl group. Explanations that attribute NEIL1 discrimination between 5fC and 5caC to properties of the substrates alone have to be reconciled with equally fast or faster excision of 5fC compared to 5caC by TDG. Note however that the activities of TDG against 5fC and 5caC containing substrates have been attributed to different mechanisms^45^. Our finding that NEIL1 distinguishes between the two types of substrates supports this conclusion.

### More extensive cooperation of NEIL1 and TDG than previously known

Niehrs and coworkers have already shown that addition of NEIL1 to TDG enhances the rate of 5fC and and 5caC excision. This finding pointed to a stimulation of NEIL1 glycosylase activity only based on the assumption that NEIL1 does not directly excise 5fC and 5caC from DNA. In our hands, only the former, but not the latter is true. Nevertheless, our data support the conclusion that NEIL1 stimulates the glycosylase activity of TDG, because the effect is retained using either glycosylase or glycosylase and lysase defective mutants of TDG. Interestingly, the enhancement of TDG activity by addition of NEIL1 is substrate specific. The effect occurs for DNA with 5fC and 5caC bases, despite differences in the mechanisms for TDG excision of 5fC and 5caC^45^. In contrast, NEIL1 mediated stimulation of TDG does not occur for the T/G mismatch substrate. In the conditions of the assay, inhibition is seen instead.

Stimulation between TDG and NEIL1 is only partially reciprocal. The glycosylase activity of NEIL1 against DNA containing 5caC or 5OHC is not affected by TDGcd. Stimulation of the glycosylase activity of NEIL1 against DNA containing 5caC is also not required biologically. As NEIL1 is a much weaker glycosylase than TDG for 5caC containing DNA, direct 5caC excision activity of NEIL1 is not required in the presence of TDG. Using TDGcd, we observed clear simulation of the AP-lyase activity of NEIL1. However, the significance of this observation is unclear for several reasons. As NEIL1 lyase rates are higher than TDG 5fC or 5caC excision rates, not much would be gained by speeding up a reaction step that appears not to be rate limiting. Moreover, the stimulation is not clearly seen with full-length TDG.

### Co-occurrence of NEIL1 and TDG in different tissues

TDG is widely expressed in different tissues according to the human protein atlas^47^, presumably to match the wide tissue distribution of 5mC. NEIL1, in line with its role in repair of oxidation damage, shows a more varied expression. The enzyme is more highly expressed in metabolically active tissues, such as brain and liver, but also in kidney. Some expression is detected in essentially any tissue^47^. Even though NEIL1 overall abundance may not reflect the nuclear abundance of the enzyme due to its role also in mitochondria, one can conclude that there is wide overlap between the expression of TDG and NEIL1, and hence there is opportunity for cooperation between the two enzymes as far as tissue distribution is concerned, despite different regulation by the cell cycle^20^.

### A model of NEIL1-TDG interactions at the replication fork

5fC and 5caC share many features with damaged DNA bases^7^. A block of Polδ or Polε by 5fC or 5caC has not yet been directly demonstrated. However, detrimental effects of 5fC and 5caC on transcription^48, 49^ make polymerase stalling at 5fC or 5caC sites very likely. We envisage two possible scenarios for the replication fork upon encounter of a 5fC or 5caC site. Replisome associated NEIL1^32^ may encounter a 5fC or 5caC site, in the absence of S-phase depleted TDG. In this case, the direct activity of NEIL1 on 5caC containing DNA could resolve the block at least for 5caC. Alternatively, replisome associated NEIL1 may encounter a 5fC or 5caC site with a TDG still bound (that has escaped the widespread TDG degradation prior to S-phase). In this scenario, the stimulation of TDG activity by NEIL1, and displacement of TDG by NEIL1, would help resolve the replication block. The substrate specific enhancement of TDG glycosylase activity reported here makes sense in this scenario: NEIL1 only stimulates TDG excision of bases (5fC and 5caC) that can be unambiguously identified as damage, but not for excision of T opposite G, which may be an incorrect choice in S-phase.

### Cooperation of TDG and NEIL1 does not lead to related phenotypes

The simplest model of TDG and NEIL1 cooperation suggests similar null phenotypes. In reality, the null phenotypes are very different. This would be expected, if TDG and NEIL1 were essential because of their respective roles in the repair of damage by deamination and by non-enzymatic oxidation. However, in the case of TDG, there is substantial evidence that the phenotype is associated with defects in DNA demethylation^18^. Even if phenotypes were completely due to demethylation defects and not due to defective repair of oxidative and deaminative lesions, the model of NEIL1-TDG cooperation envisages so different roles of the two enzymes in DNA demethylation that the different phenotypes are not surprising. Whether NEIL1 activity on 5caC containing DNA, or NEIL1-TDG interactions play any role in the NEIL1 phenotype remains to be understood.

## Methods

### DNA oligonucleotides

Chemically synthesised and HPLC purified 41-mer DNA oligonucleotides containing different base modifications at the position 20 in case of the top strand and position 22 (opposite position 20 in the top strand) in case of the lower strand were purchased from Purimex (Germany). The sequence of the top strand oligos was 5′-GCTACCTACCTAGCAGGGGXCAGCTGTCCCACTGCTCGGAA-3′, where X stands for C (cytosine), 5OHC (5-hydroxycytosine), 5hmC (5-hydroxymethylcytosine), 5fC (5-formylcytosine), 5caC (5-carboxylcytosine) or T (thymine) was radioactively labelled at the 5′ end using ^32^P using T4 polynucleotide kinase (PNK, ThermoFisher). The labelled oligos were purified using MiniQuick Spin DNA columns (Roche) according to manufacturer′s protocol. All of the top strand oligos (containing C, 5OHC, 5hmC, 5fC, 5caC and T) were additionally purified using 20% Urea-PAGE. The sequence of the bottom strand oligos was 5′-TTCCGAGCAGTGGGACAGCTG**G**CCCCTGCTAGGTAGGTAGC-3′ or 5′- TTCCGAGCAGTGGGAC**CAAGTTAAAAG**GCTAGGTAGGTAGC-3′ (Genomed), where bases in bold indicate the guanine paired with the X-base or an inserted, non-complementary region. dsDNA or DNA with a bubble structure were formed by slow annealing.

### Expression vectors and molecular cloning

NEIL1: pET28 plasmid encoding full-length wild-type human NEIL1 with C-terminal hexahistidine tag (hereinafter NEIL1 wt) was obtained from Prof Barbara Tudek and Prof Bertrand Castaing. The NEIL1 P2T, E3Q, R242A, R257A, R242A/R257A variants were prepared by PCR using specific primers containing one nucleotide mutation and PfuPlus! high fidelity thermostable polymerase (Eurx) according to manufacturer’s instructions. Reaction mixtures were supplemented with 0.01 mg/ml DpnI and incubated overnight at 37 °C. Plasmids containing the mutation were amplified in *E. coli* Top10 cells and the presence of the mutation was confirmed by sequencing (Genomed).

TDG: pET28c(+) plasmid encoding full-length wild-type human TDG with N-terminal hexahistidine tag was a kind gift from Primo Schär (Addgene #70758). The catalytic domain of TDG (TDGcd, amino acids 82–308) was prepared as follows. The region of TDG nucleotide sequence corresponding to amino acid residues 82–308 was amplified by PCR from the above plasmid using PfuPlus! polymerase (Eurx). The insert and an in-house altered version of the pET28 vector were treated with SacI FD and NotI FD restrictases (ThermoFisher), independently gel-purified, and ligated using T4 Ligase (ThermoFisher). The new construct contained an N-terminal 6x-His tag connected to serine 82 of TDG by an SSGLS linker. Plasmids containing the insert were amplified in *E. coli* Top10 cells and the presence of the mutation was confirmed by sequencing (Genomed). The catalytically inactive mutant of TDGcd (TDGcdN140A) was made by PCR protocol similar to the one used to prepare NEIL1 mutants.

### Recombinant protein expression purification

NEIL1 and variants: pET28 plasmids encoding C-terminally hexahistidine-tagged NEIL1 or variants were transferred into *E. coli* BL21-RIL (DE3) cells. A single colony from the Petri dish was used to produce an overnight starter culture. LB medium containing chloramphenicol (Cm) and kanamycin (Km) was inoculated with starter culture in 1:200 ratio and grown at 37 °C and 150 rpm shaking till the OD 0.6. The culture was then chilled to room temperature, and overnight protein expression at 30 °C was induced by 0.5 mM IPTG addition (shaker setting 150 rpm). Lower temperature, long expression time and mild induction conditions were chosen according to literature recommendations^50, 51^. Cells were collected by 20 min centrifugation at 4000 g. Cell pellets were suspended in ice-cold buffer A (50 mM Na phosphate pH 8.0, 300 mM NaCl, 10 mM imidazole, 10 mM β-mercaptoethanol) and lysed in using French press at 22kpsi. Immediately after lysis 1 mM PMSF was added and lysates were clarified by centrifugation at 35000 g for 30 min. Supernatant from 2 litres of culture was applied on disposable column packed with 5 ml of Ni-NTA sepharose (Qiagen) and pre-equilibrated with buffer A. After sample application the column was washed with 100 ml of buffer A, 100 ml of buffer B1 (50 mM Na phosphate pH 8.0, 300 mM NaCl, 20 mM imidazole, 10 mM β-mercaptoethanol), 100 ml of buffer B2 (50 mM Na phosphate pH 8.0, 100 mM NaCl, 20 mM imidazole, 10 mM β-mercaptoethanol) and eluted with 100 ml buffer C (50 mM Na phosphate pH 8.0, 300 mM NaCl, 250 mM imidazole, 10 mM β-mercaptoethanol). All the fractions were analysed by SDS-PAGE and fractions containing NEIL1 protein were pooled and diluted in 1:1.1 ratio with buffer D (50 mM Na phosphate pH 8.0, 5 mM NaCl, 10 mM β-mercaptoethanol). This protein solution was applied at flow rate 1 ml/min on the HiTrap Heparin 5 ml column (GE Healthcare), pre-equilibrated with buffer E (50 mM Na phosphate pH 8.0, 50 mM NaCl, 10 mM β-mercaptoethanol) and eluted with 50–800 linear NaCl gradient in 60 ml. The NEIL1 fractions were pooled and dialysed against buffer F (20 mM Tris-HCl pH7.5, 100 mM KCl, 2 mM DTT, 0.2 mM EDTA) overnight at +4 °C. The dialysed protein solution was mixed with anhydrous glycerol in 1:1 ratio and stored at −20 °C.

TDG: pET28c(+) encoding N-terminally hexahistidine-tagged TDG or its variants was introduced into *E. coli* BL21-RIL (DE3) cells. A single colony from Petri dish was used to produce an overnight starter culture. LB media containing Cm and Km were inoculated with starter cultures in 1:200 ratio and grown at 37 °C and 150 rpm shaking till the OD 0.6. Cultures were then cooled to room temperature, and protein expression was induced by adding 0.1 mM IPTG to the culture and incubating overnight at 25 °C (shaker setting 160 rpm). The protocol of TDG purification was optimised from the original Primo Schär protocol^52^. In detail, cells were collected by centrifugation 20 min at 4000 g. Cell pellets were suspended in ice-cold buffer 1 (50 mM Na phosphate pH 8.0, 750 mM NaCl, 20% glycerol, 1 mM imidazole, 10 mM β-mercaptoethanol) in and lysed in using French press at 22kpsi. Immediately after lysis 1 mM PMSF was added and lysates were clarified by centrifugation at 35000 g for 30 min. Supernatant was applied on HisTrap 5 ml (GE Healthcare) pre-equilibrated with buffer 1 at 1 ml/min flow rate. After sample application column was washed with 100 ml of buffer 1 and 100 ml of buffer 2 (50 mM Na phosphate pH 8.0, 750 mM NaCl, 20% glycerol, 20 mM imidazole, 10 mM β-mercaptoethanol). TDG was eluted with 20–250 mM linear imidazole gradient. Fractions containing TDG were pooled and diluted with buffer 3 (50 mM Na phosphate pH 8.0, 5 mM NaCl, 10% glycerol, 10 mM β-mercaptoethanol) in 1:15 ratio. Diluted protein prep was applied on HiTrap Heparin 5 ml (GE Healthcare) pre-equilibrated with buffer 4 (50 mM Na phosphate pH 8.0, 50 mM NaCl, 10% glycerol, 10 mM β-mercaptoethanol) at flow rate 1 ml/min. The column was washed with 150 ml of buffer 4 and TDG was eluted by 50–800 mM NaCl gradient in 60 ml. TDG fractions were pooled and dialysed overnight against buffer 5 (50 mM Na phosphate pH 8.0, 100 mM NaCl, 10% glycerol, 10 mM β-mercaptoethanol). This protein solution was diluted with anhydrous glycerol in 1:1 ratio and stored at −20 °C.

The purity of proteins used in this study was checked by SDS-PAGE (Suppl. Fig. 1).

### Glycosylase assay

For the visualisation of the enzymatic activity glycosylases NEIL1 and TDG were mixed with ^32^P labelled single-stranded or double-stranded DNA oligonucleotides in 10 μl reaction volume containing reaction buffer (70 mM MOPS, pH7.5, 5%glycerol, 1 mM EDTA and 1 mM DTT). Glycosylases were used in the concentration range 10–200 nM and oligonucleotides were used in the concentration range 10–20 nM. After incubation at 37 °C reactions were stopped by addition of an equal volume of 2x loading solution (96% formamide, 20 mM EDTA, 0.1% bromophenol blue) and incubation for 5 min at 95 °C. Samples were optionally supplemented with 0.1 M NaOH and incubated for 5 min at 95 °C to break DNA strands at abasic sites prior addition of loading solution. Reactions were resolved on 20% Urea-PAGE gels pre-heated to approx. 60 °C. Radioactively labelled DNA was detected using Typhoon Trio (GE Healthcare) or Storm (Amersham) phosphorimagers. Images were processed using the ImageQuant (gel format to bmp), Gimp (bmp to tiff, resolution definition) and MS PowerPoint (labelling).

### Lyase assay

In order to generate apyrimidinic site (AP-site), 5′ ^32^P labelled 5fC containing 41mer double-stranded oligonucleotide was mixed with 10x molar excess of TDG in reaction buffer and incubated for 1 hour at 37 °C. After the incubation the reaction was mixed 1:1 with phenol-chloroform-isoamyl alcohol mixture (25:24:1, Sigma), vortexed for 1 min and centrifuged 2 min at 12000 g. DNA containing water fraction was washed with chloroform and supplemented with 3 volumes of ethanol. The mixture was kept at −20 °C for 30 min and then centrifuged 20 min at 15000 g. The DNA pellet was dehydrated at room temperature and then resuspended in 10 mM Tris-HCl, pH 8.0. AP-site containing oligonucleotides were then treated with NEIL1 and TDG proteins and analysed as it is shown in glycosylase assay, except for shortening the incubation time with loading solution to 2½ min in order to minimize spontaneous strand breaks. Alkaline treatment was avoided, except for the positive controls.

### Electrophoretic mobility shift assay

^32^P labelled double-stranded DNA oligonucleotides containing C, 5OHC and 5hmC at concentration 10 nM were mixed with growing concentrations of NEIL1 P2T (1–200 nM) in 20 μl of the reaction volume, supplemented with 25 mM Tris-HCl, pH7.5, 100 mM NaCl and 1 mM dithiothreitol. Reaction mixtures were incubated on ice for 30 min, were supplemented with 4 μl of 6x native loading solution (10 mM Tris-HCl, pH 7.6, 60% glycerol, 0,03% bromophenol blue, 60 mM EDTA) and immediately loaded on the 6% native PAGE. Electrophoresis was done using chilled to 4 °C 0.5xTBE as a running buffer and constant voltage (100 V). Resolved radioactively labelled DNA was detected using Typhoon Trio phoshorimager (GE Healthcare).

### Size exclusion chromatography

Physical interaction between NEIL1 and TDG was tested by size-exclusion chromatography using a Superdex 200 10/300 GL column and ÄKTA Purifier FPLC system (GE Healthcare). Both NEIL1 E3Q and full-length TDG wt were dialyzed overnight against 250-fold larger volume of SEC buffer (70 mM MOPS, pH7.5, 5%glycerol, 1 mM EDTA, 1 mM DTT and 100 mM KCl). Samples containing 5nmoles of either NEIL1 or TDG or 5nmoles of both in 0.5 ml total volume were incubated for 30 min on ice and applied on the column pre-equilibrated with SEC buffer and resolved at 0.4 ml flow rate. As molecular mass standards, we used bovine thyroglobulin (669 kDa), bovine albumin fraction V (66 kDa), albumin from chicken egg white (44.3 kDa), equine myoglobin (17.6 kDa) and vitamin B12 (1.36 kDa). Albumin fraction V was from Carl Roth, all other standards were purchased from Sigma Aldrich.

All data generated or analysed during this study are included in this published article (and its Supplementary Information files).

## Electronic supplementary material

Supplementary information

## References

[1] Franchini DM (2014). Processive DNA demethylation via DNA deaminase-induced lesion resolution. PLoS One.

[2] Santos F (2013). Active demethylation in mouse zygotes involves cytosine deamination and base excision repair. Epigenetics Chromatin.

[3] Tahiliani M (2009). Conversion of 5-methylcytosine to 5-hydroxymethylcytosine in mammalian DNA by MLL partner TET1. Science.

[4] Ito S (2011). Tet proteins can convert 5-methylcytosine to 5-formylcytosine and 5-carboxylcytosine. Science.

[5] Wu SC, Zhang Y (2010). Active DNA demethylation: many roads lead to Rome. Nat Rev Mol Cell Biol.

[6] Gehring M, Reik W, Henikoff S (2009). DNA demethylation by DNA repair. Trends Genet.

[7] Bochtler M, Kolano A, Xu GL (2017). DNA demethylation pathways: Additional players and regulators. Bioessays.

[8] Schmitz KM (2009). TAF12 recruits Gadd45a and the nucleotide excision repair complex to the promoter of rRNA genes leading to active DNA demethylation. Mol Cell.

[9] Grin I, Ishchenko AA (2016). An interplay of the base excision repair and mismatch repair pathways in active DNA demethylation. Nucleic Acids Res.

[10] He Y-F (2011). Tet-mediated formation of 5-carboxylcytosine and its excision by TDG in mammalian DNA. Science.

[11] Zhu J-K (2009). Active DNA demethylation mediated by DNA glycosylases. Annu Rev Genet.

[12] Krokan HE, Drablos F, Slupphaug G (2002). Uracil in DNA–occurrence, consequences and repair. Oncogene.

[13] Hendrich B, Hardeland U, Ng HH, Jiricny J, Bird A (1999). The thymine glycosylase MBD4 can bind to the product of deamination at methylated CpG sites. Nature.

[14] Spruijt CG (2013). Dynamic readers for 5-(hydroxy)methylcytosine and its oxidized derivatives. Cell.

[15] Maiti A, Drohat AC (2011). Thymine DNA glycosylase can rapidly excise 5-formylcytosine and 5-carboxylcytosine: potential implications for active demethylation of CpG sites. J Biol Chem.

[16] Neddermann P (1996). Cloning and expression of human G/T mismatch-specific thymine-DNA glycosylase. J Biol Chem.

[17] Neddermann P, Jiricny J (1993). The purification of a mismatch-specific thymine-DNA glycosylase from HeLa cells. J Biol Chem.

[18] Cortazar D (2011). Embryonic lethal phenotype reveals a function of TDG in maintaining epigenetic stability. Nature.

[19] Cortellino S (2011). Thymine DNA glycosylase is essential for active DNA demethylation by linked deamination-base excision repair. Cell.

[20] Hardeland U, Kunz C, Focke F, Szadkowski M, Schar P (2007). Cell cycle regulation as a mechanism for functional separation of the apparently redundant uracil DNA glycosylases TDG and UNG2. Nucleic Acids Res.

[21] Slenn TJ (2014). Thymine DNA glycosylase is a CRL4Cdt2 substrate. J Biol Chem.

[22] Shibata E, Dar A, Dutta A (2014). CRL4Cdt2 E3 ubiquitin ligase and proliferating cell nuclear antigen (PCNA) cooperate to degrade thymine DNA glycosylase in S phase. J Biol Chem.

[23] Muller U, Bauer C, Siegl M, Rottach A, Leonhardt H (2014). TET-mediated oxidation of methylcytosine causes TDG or NEIL glycosylase dependent gene reactivation. Nucleic Acids Res.

[24] Hu X (2014). Tet and TDG mediate DNA demethylation essential for mesenchymal-to-epithelial transition in somatic cell reprogramming. Cell Stem Cell.

[25] Guo F (2014). Active and passive demethylation of male and female pronuclear DNA in the mammalian zygote. Cell Stem Cell.

[26] Xue JH (2016). Uracil-DNA Glycosylase UNG Promotes Tet-mediated DNA Demethylation. J Biol Chem.

[27] Vik ES (2012). Biochemical mapping of human NEIL1 DNA glycosylase and AP lyase activities. DNA Repair (Amst).

[28] Doublie S, Bandaru V, Bond JP, Wallace SS (2004). The crystal structure of human endonuclease VIII-like 1 (NEIL1) reveals a zincless finger motif required for glycosylase activity. Proc Natl Acad Sci USA.

[29] Zhu C (2016). Tautomerization-dependent recognition and excision of oxidation damage in base-excision DNA repair. Proc Natl Acad Sci USA.

[30] Burgess S, Jaruga P, Dodson ML, Dizdaroglu M, Lloyd RS (2002). Determination of active site residues in Escherichia coli endonuclease VIII. J Biol Chem.

[31] Bandaru V, Sunkara S, Wallace SS, Bond JP (2002). A novel human DNA glycosylase that removes oxidative DNA damage and is homologous to Escherichia coli endonuclease VIII. DNA Repair (Amst).

[32] Hegde ML (2013). Prereplicative repair of oxidized bases in the human genome is mediated by NEIL1 DNA glycosylase together with replication proteins. Proc Natl Acad Sci USA.

[33] Hegde PM (2015). The C-terminal Domain (CTD) of Human DNA Glycosylase NEIL1 Is Required for Forming BERosome Repair Complex with DNA Replication Proteins at the Replicating Genome: DOMINANT NEGATIVE FUNCTION OF THE CTD. J Biol Chem.

[34] Dou H (2008). Interaction of the human DNA glycosylase NEIL1 with proliferating cell nuclear antigen. The potential for replication-associated repair of oxidized bases in mammalian genomes. J Biol Chem.

[35] Vartanian V (2006). The metabolic syndrome resulting from a knockout of the NEIL1 DNA glycosylase. Proc Natl Acad Sci USA.

[36] Nemec AA, Wallace SS, Sweasy JB (2010). Variant base excision repair proteins: contributors to genomic instability. Semin Cancer Biol.

[37] Yeo J, Goodman RA, Schirle NT, David SS, Beal PA (2010). RNA editing changes the lesion specificity for the DNA repair enzyme NEIL1. Proc Natl Acad Sci USA.

[38] Niehrs C, Schafer A (2012). Active DNA demethylation by Gadd45 and DNA repair. Trends Cell Biol.

[39] Schomacher L (2016). Neil DNA glycosylases promote substrate turnover by Tdg during DNA demethylation. Nat Struct Mol Biol.

[40] Coey CT (2016). Structural basis of damage recognition by thymine DNA glycosylase: Key roles for N-terminal residues. Nucleic Acids Res.

[41] Hardeland U, Steinacher R, Jiricny J, Schar P (2002). Modification of the human thymine-DNA glycosylase by ubiquitin-like proteins facilitates enzymatic turnover. Embo J.

[42] Vartanian V (2017). NEIL1 protects against aflatoxin-induced hepatocellular carcinoma in mice. Proc Natl Acad Sci USA.

[43] Zhang QM (2005). DNA glycosylase activities for thymine residues oxidized in the methyl group are functions of the hNEIL1 and hNTH1 enzymes in human cells. DNA Repair (Amst).

[44] Imamura K, Averill A, Wallace SS, Doublie S (2012). Structural characterization of viral ortholog of human DNA glycosylase NEIL1 bound to thymine glycol or 5-hydroxyuracil-containing DNA. J Biol Chem.

[45] Maiti A, Michelson AZ, Armwood CJ, Lee JK, Drohat AC (2013). Divergent mechanisms for enzymatic excision of 5-formylcytosine and 5-carboxylcytosine from DNA. J Am Chem Soc.

[46] Dai Q (2016). Weakened N3 Hydrogen Bonding by 5-Formylcytosine and 5-Carboxylcytosine Reduces Their Base-Pairing Stability. ACS Chem Biol.

[47] Uhlen M (2015). Proteomics. Tissue-based map of the human proteome. Science.

[48] Fousteri M, Mullenders LHF (2008). Transcription-coupled nucleotide excision repair in mammalian cells: molecular mechanisms and biological effects. Cell Res.

[49] Kellinger MW (2012). 5-formylcytosine and 5-carboxylcytosine reduce the rate and substrate specificity of RNA polymerase II transcription. Nat Struct Mol Biol.

[50] Jhamb K, Sahoo DK (2012). Production of soluble recombinant proteins in Escherichia coli: effects of process conditions and chaperone co-expression on cell growth and production of xylanase. Bioresour Technol.

[51] Schein CH (1989). Production of Soluble Recombinant Proteins in Bacteria. Bio-Technol.

[52] Weber AR (2016). Biochemical reconstitution of TET1-TDG-BER-dependent active DNA demethylation reveals a highly coordinated mechanism. Nat Commun.

[53] Zhang L (2012). Thymine DNA glycosylase specifically recognizes 5-carboxylcytosine-modified DNA. Nat Chem Biol.

