# Supplementary material for: Nei-like 1 (NEIL1) excises 5-carboxylcytosine directly and stimulates TDG-mediated 5-formyl and 5-carboxylcytosine excision
Source: Sci Rep. 2017 Aug 21;7:9001. doi: 10.1038/s41598-017-07458-4 (PMC5566547; doi:10.1038/s41598-017-07458-4)
Supplement: Supplementary file 1 — Supplementary information [file 41598_2017_7458_MOESM1_ESM.pdf]

Supplementary information for:

Nei-like 1 (NEIL1) excises 5-carboxylcytosine directly and stimulates TDG-mediated 5-formyl and 5-carboxylcytosine excision

Anton Slyvka, Karolina Mierzejewska, Matthias Bochtler

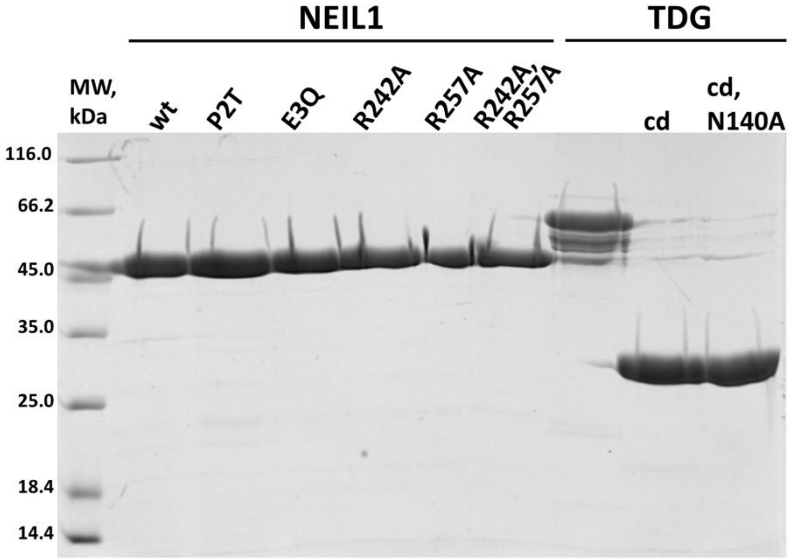

Suppl. Fig. 1: Purity of proteins used in this study: analysed by denaturing 12% SDS-PAGE electrophoresis. The gel was stained using Coomassie Brilliant Blue.

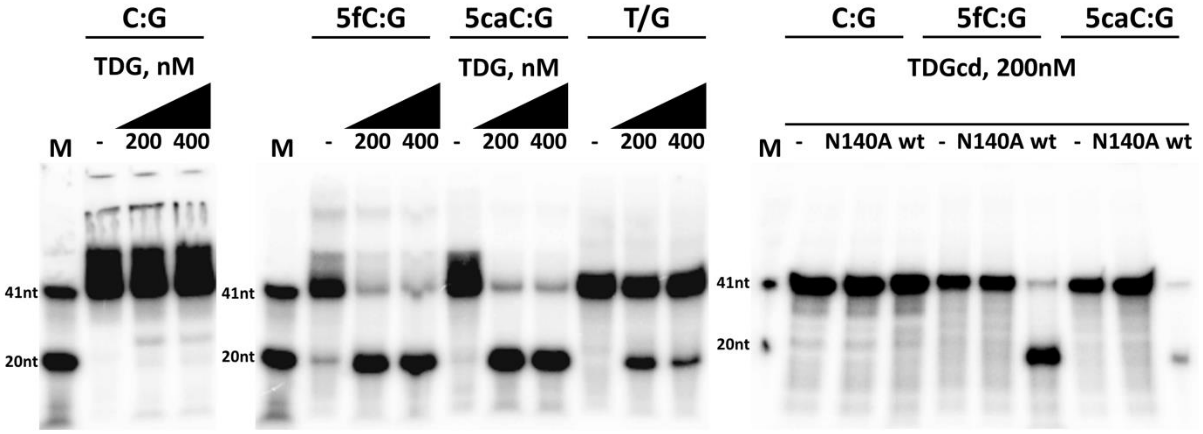

Suppl. Fig. 2: Activity of TDG or TDGcd against 5caC/G, 5fC/G and T/G containing substrates: Indicated concentrations of full-length TDG or TDGcd were incubated (37°C, 60min) with 20nM dsDNA containing 5fC, 5caC, or T opposite G. Reaction products were subjected to alkaline conditions to break DNA strands at AP-sites. The experiment confirms the known TDG glycosylase activities for our preparations of the full length enzyme and the catalytic domain.

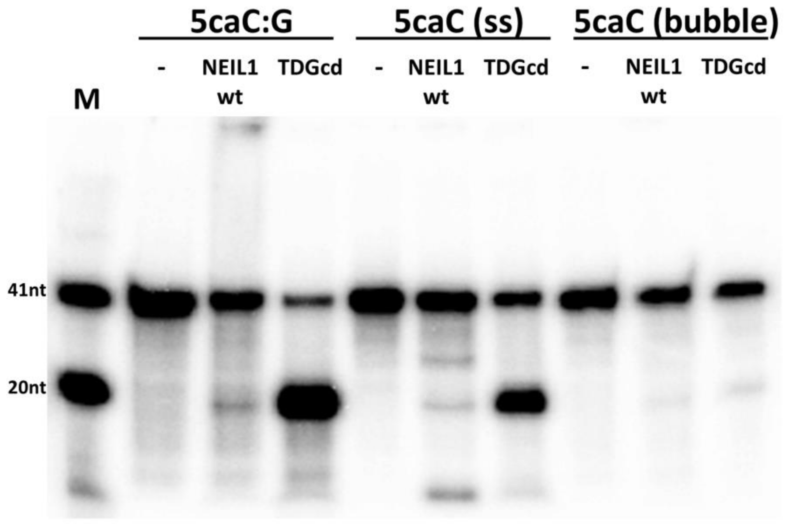

Suppl. Fig. 3: Glycosylase activities of TDG and NEIL1 against DNA containing 5caC in different structural contexts: TDGcd (200nM) and NEIL1 (200nM) were incubated (37°C, 60min) with a radiolabelled DNA strand with 5caC at position 20 (20nM), presented either in the context of dsDNA, as a single strand, or in a bubble structure. DNA was then broken by alkaline treatment at AP-sites. Glycosylase activity was detected in all contexts. Note that TDG is the much more active glycosylase, independent of structural context.

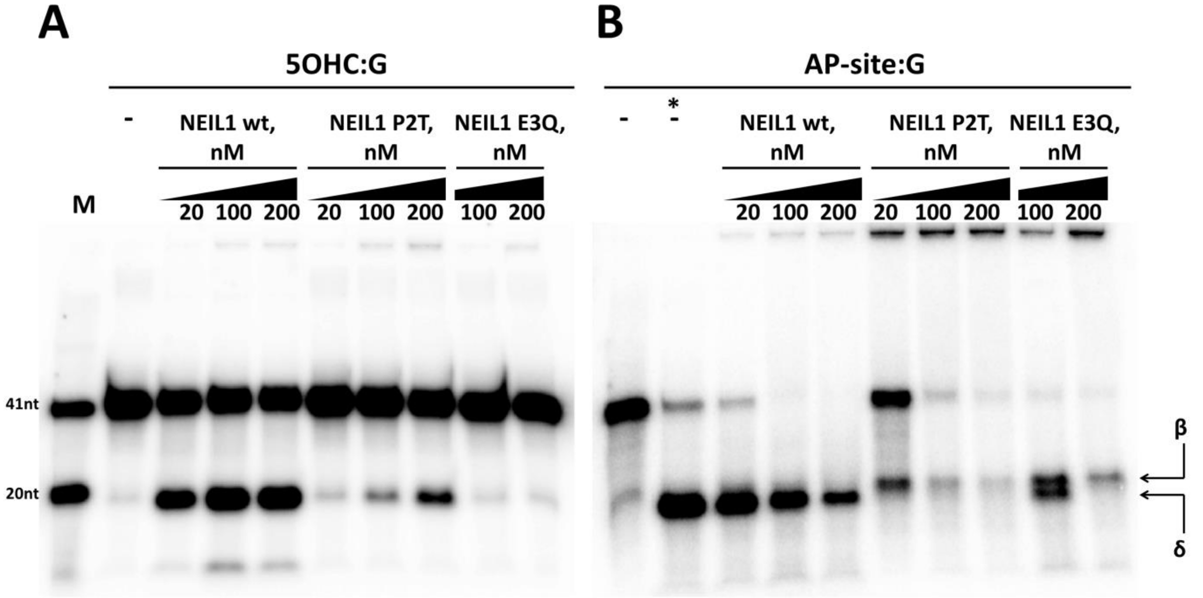

Suppl. Fig. 4: Effect of P2T and E3Q substitutions on NEIL1 activity: NEIL1 and variants were incubated (37°C, 60min) with 20nM radiolabeled dsDNA substrate containing a 5OHC base (A) or an AP-site (B). In the experiment with 5OHC containing DNA, but not experiment with AP-site containing DNA, AP-sites were chemically converted to single strand breaks in alkaline conditions after the reaction. The experiment using the 5OHC containing substrate measures the glycosylase activities of NEIL1 or NEIL1 variants independently of the lyase activities. Conversely, the experiment using the AP-site substrate measures the lyase activities of NEIL1 and variants

independently of the glycosylase activities. NEIL1 variants are large inactive. Residual activities are similar to those of the equivalent variants of the *E. coli* enzyme. Note that for higher NEIL1 concentration, some of the DNA stays in the well of the gel. ‘*’ represents the AP-site containing dsDNA treated with alkaline conditions (positive control). ‘β’ and ‘δ’ mark the products of β- and δ-lyase activity of NEIL1 respectively.

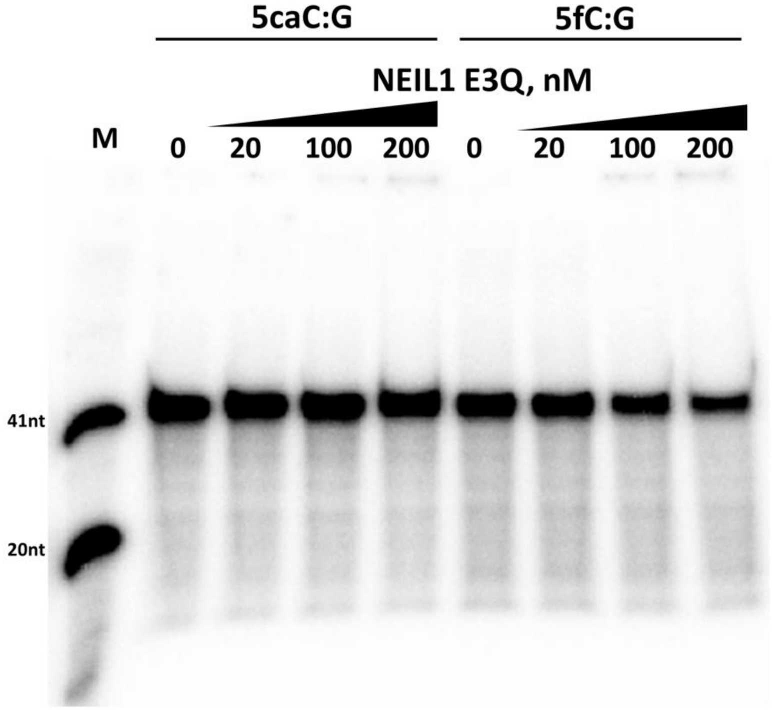

Suppl. Fig. 5: Lack glycosylase activity of E3Q on dsDNA containing 5fC or 5caC: Growing concentrations NEIL1 E3Q were incubated (37°C, 60min) with dsDNA containing a radiolabeled strand with either 5caC or 5fC. Autoradiography after work-up in alkaline conditions and denaturing gel electrophoresis showed no indication of glycosylase activity of the mutant against either DNA.

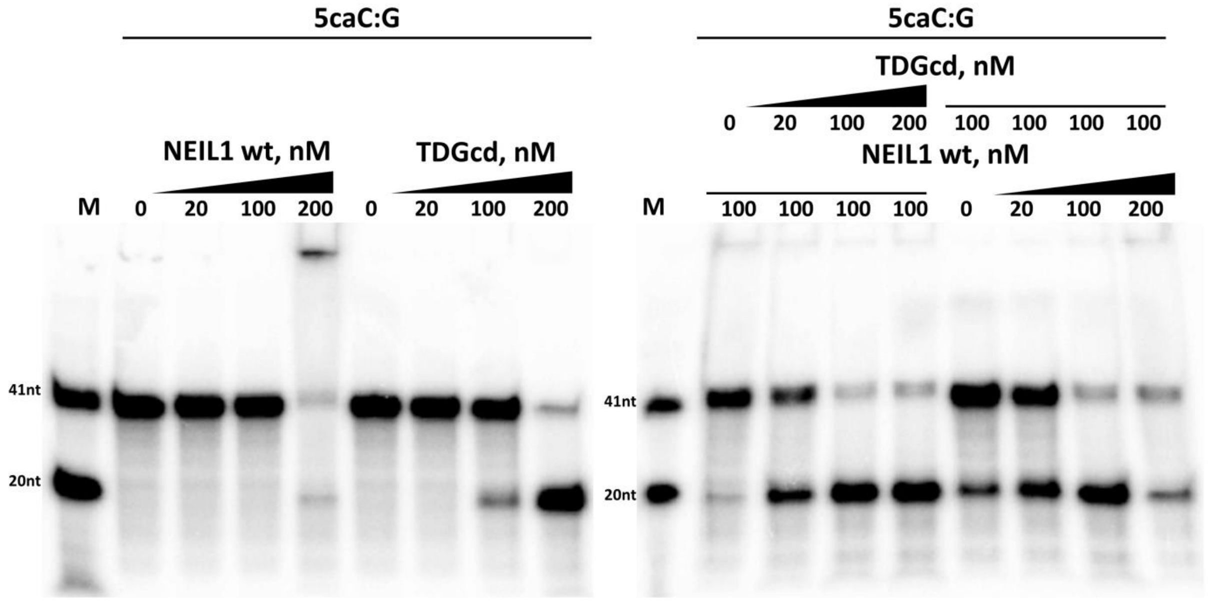

Suppl. Fig. 6: Glycosylase activities of NEIL1 and TDGcd alone and in combination against a 5caC containing dsDNA substrate: TDGcd or NEIL1 were incubated separately or in combination with 20nM dsDNA containing 5caC opposite G. The experiment confirms that addition of NEIL1 to TDG and vice versa enhances glycosylase activity on DNA containing 5caC. In the case of NEIL1 addition to a fixed amount of TDG, the rate enhancement is larger than can be explained by the glycosylase activity of NEIL1 alone, suggesting that the glycosylase activity of at least one of the two proteins must be stimulated by interaction with the other.

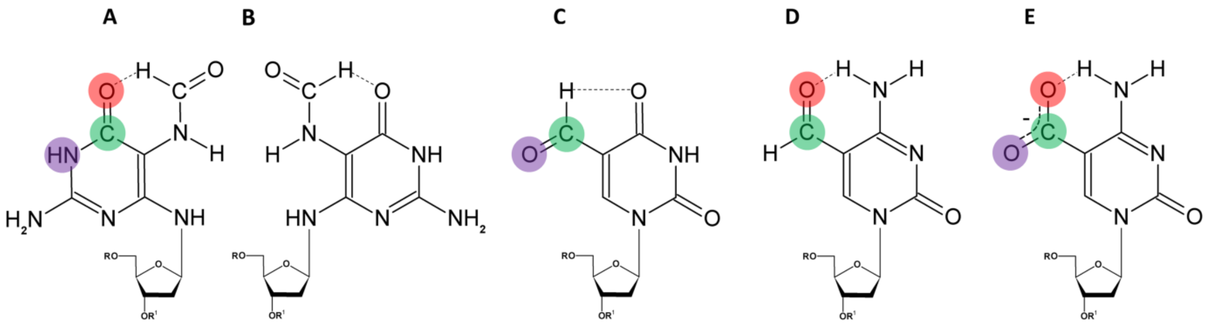

Suppl. Fig. 7: Plausibility of NEIL1 activity on 5caC containing substrates: Comparison of bases in some established DNA substrates of NEIL1 (A-C) with 5fC and 5caC. (A,B) 2’-deoxynucleoside of 2,6-diamino-4-hydroxy-5-formamidopyrimidine (FapyG, derived from 8-oxoguanine ring opening, shown in syn- and anti-conformations) (C) 2’-deoxy-5-formyluridine (D) 2’-deoxy-5-formylcytidine (E) 2’-deoxy-5-carboxylcytidine.
